# Performance comparison of two-point linkage methods using microsatellite markers flanking known disease locations

**DOI:** 10.1186/1471-2156-6-S1-S141

**Published:** 2005-12-30

**Authors:** Mark W Logue, Andrew W George, M Anne Spence, Veronica J Vieland

**Affiliations:** 1 Center for Statistical Genetics Research, College of Public Health and the Roy J & Lucille A Carver College of Medicine, University of Iowa, Iowa City, IA, USA; 2 Program in Public Health Genetics, College of Public Health, University of Iowa, Iowa City, IA, USA; 3 Department of Pediatrics, University of California Irvine Medical Center, Orange, CA, USA; 4 Department of Psychiatry, Roy J & Lucille A Carver College of Medicine, University of Iowa, Iowa City, Iowa, USA

## Abstract

The Genetic Analysis Workshop 14 simulated data presents an interesting, challenging, and plausible example of a complex disease interaction in a dataset. This paper summarizes the ease of detection for each of the simulated Kofendrerd Personality Disorder (KPD) genes across all of the replicates for five standard linkage statistics. Using the KPD affection status, we have analyzed the microsatellite markers flanking each of the disease genes, plus an additional 2 markers that were not linked to any of the disease loci. All markers were analyzed using the following two-point linkage methods: 1) a MMLS, which is a standard admixture LOD score maximized over θ, α, and mode of inheritance, 2) a MLS calculated by GENEHUNTER, 3) the Kong and Cox LOD score as computed by MERLIN, 4) a MOD score (standard heterogeneity LOD maximized over θ, α, and a grid of genetic model parameters), and 5) the PPL, a Bayesian statistic that directly measures the strength of evidence for linkage to a marker. All of the major loci (D1–D4) were detectable with varying probabilities in the different populations. However, the modifier genes (D5 and D6) were difficult to detect, with similar distributions under the null and alternative across populations and statistics. The pooling of the four datasets in each replicate (*n *= 350 pedigrees) greatly improved the chance of detecting the major genes using all five methods, but failed to increase the chance to detect D5 and D6.

## Background

In this study we used the simulated the Genetic Analysis Workshop 14 (GAW14) data using the Kofendrerd Personality Disorder (KPD) affection status as our phenotype. We did this with full knowledge of the generating model. We chose to examine the performance of the statistics by comparing markers flanking a known disease gene location to a pair of markers from a chromosome containing no disease genes. Our data consist of 13 markers: two markers flanking D1, D3, D4, D5, and D6, a single marker flanking D2 (because it falls at the end of chromosome 3), and our arbitrarily chosen unlinked markers, D04S128 and D04S129, which we refer to as markers flanking unlinked locus U1. We analyzed the data from all 100 replicates in each of the four populations as well as creating a pooled dataset of 350 pedigrees created by combining the data from all four populations.

## Methods

### MMLS

The first statistic we examined was the maximized maximum LOD score (MMLS) [1-3] that is a standard admixture heterogeneity LOD score (HLOD) maximized over θ, α, and mode of inheritance (dominant/recessive). MMLS scores were computed using MLIP [4]. For both the dominant and the recessive model the penetrance for an individual not carrying any disease alleles was set to 1% while the penetrance for genetically affected individuals was set to 80%. The risk allele frequency assumed was 1% under the dominant model and 10% under the recessive model. Note that this differs from the MMLS reported in Hodge et al. [2], in which homogeneity and different genetic model parameters were assumed.

### MLS

Risch's maximum LOD score statistics (MLS scores) [5,6] were computed using GENEHUNTER [7], allowing for dominance variance. GENEHUNTER was run, discarding the unaffected individuals. A max-bits setting of 24 was used for all datasets except for replicate 43 of the NY data, which would not finish unless the max-bits was set to 22. All pairs were used with unequal weight to reflect the appropriate per-pedigree influence. Note that GENEHUNTER estimates the identical-by-descent (IBD) sharing under the triangle constraint.

### Kong and Cox LOD scores

Two-point Kong and Cox LOD scores (KCLS) [8], were computed using MERLIN's [9] single-point option. A max-bits setting of 50 was used, which caused 26 pedigrees (across all replicates) to be dropped from the analysis. Specifically, 22 replicates of the NY dataset had one pedigree that exceeded 50 bits and two replicates of the NY dataset had two pedigrees which exceeded 50 bits. No replicates had more than two pedigrees that exceeded 50 bits.

### PPL

We examined the performance of a Bayesian statistic, the posterior probability of linkage (PPL) [10-13]. The PPL directly measures the probability that a disease gene is linked to a particular marker (or genomic location in the multipoint case). The PPL incorporates an unknown genetic model by placing priors on the elements of the genetic model and integrating them out of the likelihood [14-16]. We present the results for the PPL in the combined dataset in two ways. First, the PPL-p, which is simply the PPL computed for the entire dataset, and second, the PPL-seq, which is the PPL computed for the entire dataset by sequentially updating across all 4 populations, using the posterior distribution of the recombination fraction, θ, from one analysis as the prior distribution for the next analysis.

### MOD

Finally, we present the results of the MOD [17] score, which is a standard admixture LOD score (HLOD) maximized over θ, the proportion of linked pedigrees (α), and the genetic model parameters. The MOD scores were computed using MLIP and were maximized over the same set of model parameters used to compute the PPL. Of course, maximizing over a larger portion of the space will result in MOD scores that are greater than MMLS scores for both the linked and unlinked markers.

## Results

The mean and maximum scores for flanking markers at each disease locus and each of the methods for each of the populations and the pooled data are contained in Table 1. In the interest of space, both flanking markers have been pooled into a single score for each disease locus (mean/max are across both replicates and flanking markers) except in the case of disease locus 2, which had only a single flanking marker.

In general, MMLS and MOD scores are larger than MLS and KCLS scores. However, the MMLS and MOD scores were also higher for the unlinked locus than the other two methods, so that the increase in score does not necessarily indicate an increase in power. Nonetheless, there are a few things that can be determined from Table 1. While disease loci 1–4 are relatively easy to identify, the results for loci 5 and 6 do not deviate far from their behavior under the null. Additionally, the means varied as a function of the population for each dataset. Pooling the data greatly increased the mean scores for the linked loci. This occurred despite the fact that the underlying disease mechanism varied widely from locus to locus.

Table 2 presents the value of P, which we define as the percentage of replicates in which the maximum score for one of the flanking markers exceeded the maximum value received under the null distribution, once again across replicates. P represents a rough approximation to the chance that each marker would be detected by a 0.01 size test (except for the D2 case for which P would be conservative). The method receiving the highest score at each population/locus is indicated in bold font in the tables. Perhaps surprisingly, there is no clear winner when the performance of these statistics were compared in this way. As indicated by the means, D5 and D6 were particularly difficult to detect, with no statistic/dataset combination able to achieve a P greater than 7%.

## Conclusion

We have compared the performance of five statistics, the MMLS, the MLS, the KCLS, the MOD, and the PPL, by examining markers flanking the known disease locations in the GAW14 simulated data. By computing P, which is an empirical measure of the power, we are able to compare statistics that have different scales. We find that none of the statistics emerges a clear victor, with different statistics having greater power depending on which disease locus and population were examined. However, it is surprising that the MMLS and MOD score, which make use of the entire pedigree structure (as opposed to the MLS, which uses only affected sib pairs, and KCLS, which uses affected relative pairs), and whose scores were calculated without any trimming or dropping of large pedigrees, were not able to utilize this information to their advantage in the NY population, where the sample consists of extended pedigrees. This is due to the high values of these statistics obtained under the null. The PPL performs better in the NY dataset, using data from the entire pedigree, without a similar inflation of null values. D1–D4 appear detectable, with maximum scores in the range that would indicate linkage. However, values of P were surprisingly low for these loci, especially since the maximum values under the null, presented in Table 1, would scarcely be considered adequate to conclude linkage. Pooling the samples for D1–D4 increased power to the range where linkage was consistently detectable, despite the fact that variation in the diagnostic schemes causes the genetic model to differ from dataset to dataset. Loci D5 and D6 were not readily detectable in any of the populations or in the pooled data.

## Abbreviations

GAW14: Genetic Analysis Workshop 14

HLOD: Heterogeneity LOD

IBD: Identity by descent

KCLS: Kong and Cox LOD scores

KPD: Kofendrerd Personality Disorder

MLS: Maximum LOD score statistic

MMLS: Maximized maximum LOD score

PPL: Posterior probability of linkage

## Authors' contributions

MWL performed analyses and prepared a draft of the manuscript. MWL, MAS, and VJV contributed computing resources. All authors contributed to study design and editing, and approved the final manuscript.

## Figures and Tables

**Table 1:** Mean/max for AI, DA, KY, NY, and combined populations

		MMLS	MLS	KCLS	MOD	PPL	PPL-p	PPL-seq
AI								
	D1	1.526/5.636	0.708/3.812	0.816/4.040	2.111/5.731	16.8%/98.2%		
	D2	2.815/8.440	2.210/6.241	2.034/6.600	3.693/9.152	43.3%/100.0%		
	D3	1.584/6.188	1.438/4.843	1.510/5.560	2.469/6.945	19.6%/99.8%		
	D4	1.775/6.904	1.339/5.198	1.194/5.380	2.500/7.044	19.2%/99.8%		
	D5	0.244/1.696	0.145/1.626	0.044/2.200	0.698/2.526	1.9%/14.4%		
	D6	0.238/1.636	0.122/1.407	0.013/2.280	0.646/2.609	1.8%/14.7%		
	U1	0.190/1.509	0.084/1.195	-0.037/1.230	0.575/2.419	1.6%/6.8%		
DA								
	D1	3.698/10.740	2.722/6.993	3.044/7.510	5.005/12.670	71.7%/100.0%		
	D2	3.156/8.144	3.159/8.106	3.367/8.190	4.673/10.480	64.6%/100.0%		
	D3	0.482/3.429	0.370/3.568	0.318/2.500	0.956/4.455	2.9%/68.0%		
	D4	0.451/2.830	0.382/2.869	0.291/4.160	0.914/4.565	3.1%/78.6%		
	D5	0.349/3.948	0.274/3.025	0.230/3.700	0.790/4.522	3.0%/79.3%		
	D6	0.256/2.258	0.109/1.298	-0.047/1.210	0.627/2.991	1.9%/18.8%		
	U1	0.243/1.928	0.128/1.211	0.017/1.190	0.616/2.277	1.6%/5.7%		
KA								
	D1	0.849/5.454	0.477/3.493	0.445/3.020	1.452/7.427	6.9%/99.7%		
	D2	2.136/5.953	1.694/5.267	1.539/4.350	2.812/7.775	27.6%/99.9%		
	D3	2.345/6.894	2.390/6.770	2.520/7.020	3.550/8.210	41.7%/99.9%		
	D4	3.418/10.510	2.606/7.270	2.244/6.320	4.523/11.660	51.1%/100.0%		
	D5	0.388/2.475	0.212/1.482	0.143/1.640	0.820/3.563	2.3%/24.0%		
	D6	0.276/1.972	0.160/2.080	0.031/1.850	0.638/3.094	1.9%/17.3%		
	U1	0.238/1.891	0.148/1.322	0.016/1.410	0.615/2.654	1.8%/14.2%		
NY								
	D1	1.076/5.906	0.311/2.224	0.485/3.170	1.932/6.002	11.4%/99.4%		
	D2	3.087/8.396	1.504/6.281	1.762/5.430	4.487/9.694	56.0/100.0%		
	D3	1.269/3.673	0.564/3.109	0.990/4.400	2.357/6.068	14.5%/98.5%		
	D4	0.928/3.107	0.401/2.319	0.376/2.480	1.689/4.632	4.4%/50.5%		
	D5	0.244/2.148	0.080/0.976	0.004/1.340	0.751/3.225	1.4%/9.1%		
	D6	0.270/2.213	0.119/1.694	0.065/0.950	0.812/2.804	1.6%/5.4%		
	U1	0.357/3.573	0.123/1.680	0.069/1.710	0.967/5.684	2.0%/32.3%		
Combined								
	D1	4.797/11.940	2.565/9.668	3.344/11.880	6.105/12.370		82.3%/100.0%	88.9%/100.0%
	D2	9.090/15.970	7.288/13.270	7.695/15.380	12.010/21.710		99.9%/100.0%	99.17%/100.0%
	D3	3.596/8.239	3.337/7.933	4.052/9.480	5.826/13.320		79.6%/100.0%	76.4%/100.0%
	D4	4.347/11.150	3.101/8.215	2.976/11.430	5.648/12.730		68.8%/100.0%	70.6%/100.0%
	D5	0.373/2.364	0.272/3.381	0.229/4.600	0.940/4.699		1.9%/60.0%	2.4%/77.0%
	D6	0.313/2.298	0.148/1.676	0.042/1.580	0.849/3.102		1.6%/24.6%	1.3%/21.7%
	U1	0.289/2.187	0.129/1.120	0.022/1.320	0.824/3.555		1.5%/28.4%	1.2%/25.8%

**Table 2:** P for AI, DA, KA, NY and combined populations

		MMLS	MLS	KCLS	MOD	PPL	PPL-p	PPL-seq
AI								
	D1	**60%^a^**	30%	40%	54%	57%		
	D2	**78%**	76%	72%	75%	77%		
	D3	61%	**70%**	69%	62%	65%		
	D4	**70%**	67%	55%	67%	68%		
	D5	2%	3%	3%	2%	**4%**		
	D6	2%	1%	2%	1%	**3%**		
DA								
	D1	97%	98%	98%	99%	**100%**		
	D2	76%	89%	**90%**	89%	**90%**		
	D3	4%	8%	10%	10%	**15%**		
	D4	6%	**13%^a^**	7%	11%	11%		
	D5	4%	**7%**	**7%**	6%	6%		
	D6	2%	2%	1%	4%	**5%**		
KA								
	D1	**20%^a^**	16%	15%	**20%**	16%		
	D2	54%	**58%**	49%	48%	45%		
	D3	77%	**93%**	89%	88%	83%		
	D4	89%	89%	83%	**93%**	86%		
	D5	**4%**	**4%**	1%	4%	3%		
	D6	1%	2%	**3%**	1%	1%		
NY								
	D1	6%	2%	8%	3%	**15%**		
	D2	32%	31%	43%	25%	**61%**		
	D3	4%	7%	**28%**	2%	24%		
	D4	0%	6%	**10%**	0%	2%		
	D5	**0%^a^**	**0%**	**0%**	**0%**	**0%**		
	D6	0%	**1%**	0%	0%	0%		
Combined								
	D1	97%	95%	98%	97%		96%	**100%**
	D2	**100%^a^**	**100%**	**100%**	**100%**		**100%**	**100%**
	D3	90%	99%	**100%**	97%		95%	98%
	D4	91%	**99%**	97%	92%		89%	95%
	D5	1%	**5%**	2%	1%		1%	4%
	D6	1%	**2%**	1%	0%		0%	0%

^a^Bold text indicates the method receiving the highest score at each population/locus.
